# Physiologically Based Pharmacokinetic–Pharmacodynamic-Based Quantification of Exposure–Response for Sodium Tanshinone IIA Sulfonate in Normal and Cerebral Ischemia–Reperfusion Injury Rats

**DOI:** 10.3390/biology15110827

**Published:** 2026-05-24

**Authors:** Ying Chen, Jinyao Zhang, Yongkang Zhang, Tian Qin, Weifeng Jin, Yifei Wang, Yunxiang Chen, Li Yu, Lijiang Zhang

**Affiliations:** 1 Zhejiang Key Laboratory of High-Level Biosafety and Biomedical Transformation, School of Public Health, Hangzhou Medical College, Hangzhou 311305, China; chenyingap@163.com (Y.C.);; 2 Key Laboratory of Drug Safety Evaluation and Research of Zhejiang Province, Center of Safety Evaluation and Research, Hangzhou Medical College, Hangzhou 310053, China; 3 School of Pharmaceutical Sciences, Zhejiang Chinese Medical University, Hangzhou 310053, China; 4 The Third School of Clinical Medicine (School of Rehabilitation Medicine), Zhejiang Chinese Medical University, Hangzhou 310053, China; 5 School of Basic Medical Sciences, Zhejiang Chinese Medical University, Hangzhou 310053, China

**Keywords:** sodium tanshinone IIA sulfonate, cerebral ischemia–reperfusion injury, pharmacokinetics, pharmacodynamics, PBPK-PD model, comparative study

## Abstract

Sodium tanshinone IIA sulfonate (STS) injection is clinically used to protect against cerebral ischemia–reperfusion injury (CIRI). This study aimed to establish physiologically based pharmacokinetic–pharmacodynamic (PBPK–PD) models for normal and CIRI rats and to quantitatively characterize the time–concentration–effect relationship, as well as disease-specific mechanistic differences. A middle cerebral artery occlusion rat model was established. STS was administered via the tail vein, and blood samples were collected at serial time points. High-performance liquid chromatography and enzyme-linked immunosorbent assay were used to quantify plasma STS concentrations and inflammatory markers, respectively, whereas equilibrium dialysis was performed to determine protein binding. PK-Sim and Python were used to establish a PBPK model, which was subsequently extrapolated to humans to construct PBPK–PD models. The results showed that plasma STS concentrations were consistently higher in the model rats than in normal rats. STS significantly reduced inflammatory levels in model rats, with a delayed onset of pharmacological effect. Human PBPK model simulations indicated that STS is rapidly eliminated in healthy individuals, while its elimination is reduced under pathological conditions. This study provides a robust modeling framework and methodological reference for dose optimization and prediction of clinical efficacy of STS in the treatment of CIRI.

## Simple Summary

Ischemic stroke frequently leads to cerebral ischemia–reperfusion injury (CIRI). Tanshinone IIA, derived from *Salvia miltiorrhiza* (a traditional Chinese medicinal herb), has demonstrated therapeutic efficacy in mitigating CIRI. Its clinically used formulation is sodium tanshinone IIA sulfonate (STS) injection. We established a middle cerebral artery occlusion model in male Sprague-Dawley rats to characterize the in vivo disposition of STS, including its time–concentration–effect relationship and disease-related mechanistic differences. We established a physiologically based pharmacokinetic (PBPK) model for both healthy and diseased rats to quantitatively characterize the disposition of STS, subsequently extrapolated the models to humans, and finally developed a PBPK–pharmacodynamic model. The results indicated that hepatic and renal clearance of STS was significantly lower in model rats than in normal rats. STS significantly reduced inflammatory levels in model rats, with a delayed onset of effect. This study provides a robust modeling framework and a methodological reference for optimizing STS dosing and predicting its clinical efficacy in CIRI treatment.

## 1. Introduction

**Figure 1:**
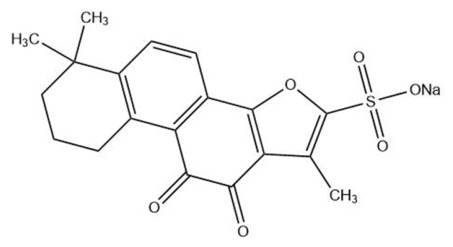
Chemical structure of sodium tanshinone IIA sulfonate (CAS: 69659-80-9).

Stroke ranks as the second leading cause of death and the third leading cause of disability worldwide, with over 12.2 million new stroke cases reported annually, of which ischemic stroke accounts for 86% of all stroke [1,2,3]. Due to its high mortality and disability rates, it imposes a heavy burden on society and the families of patients [4]. The estimated cost of stroke worldwide exceeds $890 billion, accounting for 0.66% of global GDP [5]. The disability rate increases significantly with the severity of stroke after 5 years, and about two-thirds of stroke survivors have multiple functional impairments [6]. Cerebral ischemia–reperfusion injury (CIRI) is a central pathological process in ischemic stroke. It involves oxidative stress, inflammation, apoptosis, blood–brain barrier (BBB) disruption, impaired energy metabolism, glutamate excitotoxicity, and calcium overload, all of which ultimately lead to neuronal injury or death [7,8]. Accordingly, the development of effective therapeutic agents remains a priority. *Salvia miltiorrhiza* Bge., a classic traditional Chinese medicinal herb, is widely applied in the management of cardiovascular and cerebrovascular diseases [9,10]. Its principal active ingredients include tanshinones and salvianolic acids. Among them, Tanshinone IIA (Tan IIA) possesses potent anti-inflammatory activity, while its clinical application is restricted by poor aqueous solubility [11]. Sodium tanshinone IIA sulfonate (STS), a sulfonated derivative (see Figure 1 for its chemical structure), possesses enhanced water solubility and bioavailability compared with Tan IIA [12]. STS exerts anticoagulant, anti-inflammatory and antioxidant effects. It has been clinically applied for multiple cardiovascular disorders and exhibits promising therapeutic potential against ischemic stroke [13,14]. However, its metabolic process and pharmacological effects in vivo are still unclear.

In vivo exposure–response relationship is usually quantified using pharmacokinetic–pharmacodynamic (PK-PD) modeling. The conventional PK-PD methods commonly adopted are largely empirical and driven by data with no direct physiological interpretation, thus lacking the potential to predict or extrapolate beyond the observed data [15]. On the other hand, physiologically based PK (PBPK) modeling involves consideration of parameters including tissue volume, blood flow rates, metabolism, etc. Integrating PD into the PBPK model can characterize the mechanism of drug action in vivo and improve the extrapolation of cross-species, dose and pathological state [16]. After establishing and validating a PBPK model using preclinical experiments, critical parameters including tissue volume and hepatic clearance rate can be translated into the human system through in vitro–in vivo extrapolation (IVIVE) or interspecies scaling, which can facilitate PBPK modeling in the human system [17]. Using a human PBPK model, the plasma concentration–time profile and tissue distribution can be predicted, and the effect of different dosing strategies on systemic exposure evaluated [18]. Thus, constructing a PBPK-PD model for quantitative assessment of drug action offers both theoretical significance and clinical value.

While previous studies have reported the pharmacokinetic characteristics of STS and its pharmacological activity in ischemic stroke [19,20], no research has yet combined PBPK modeling with PD models and systematically quantified the exposure effect relationship of STS in CIRI pathological states. Compared to the PBPK-PD model, the classic atrioventricular model has been mostly used for PK-PD analysis in the past, lacking clear physiological significance and making it difficult to extrapolate to different populations or disease states [21,22,23]. In this study, a pharmacokinetic model with clear physiological significance was constructed by incorporating the physiological parameters of rats and the physical and chemical properties of drugs, and extrapolated to a human model, which provided a scientific basis for STS in the design of individualized drug delivery scheme for stroke patients. However, relying solely on PD cannot accurately describe the efficacy of drugs in CIRI state. After CIRI, the integrity of the blood–brain barrier is impaired and there are significant changes in cerebral hemodynamics [24]. Research has shown that the integrity of the blood–brain barrier (BBB) is disrupted after cerebral ischemia, leading to increased permeability, pathological and physiological changes, and aggravated neuronal damage. These changes become more pronounced after blood flow recovery [25]. At the same time, the function of drug transporters may also be impaired, thereby altering the tissue distribution and clearance characteristics of drugs [26]. These lead to significant differences in the distribution of drugs between plasma and target tissues, and directly linking plasma drug concentration with anti-inflammatory effects can cause significant bias. Therefore, introducing the effect chamber, a mathematically hypothetical compartment structure [27], into the PBPK model can more effectively describe the transport and accumulation process of drugs from plasma to the central nervous system effect site, and reflect its time–concentration–effect relationship with anti-inflammatory response.

In this study, a PBPK-PD model incorporating an effect compartment was developed to characterize the exposure–response relationship of STS in both normal and CIRI model rats. The model was used to systematically and quantitatively evaluate alterations in the distribution, transport, and elimination of STS under pathological conditions and to elucidate disease-related effects on its PKs. Furthermore, the model was extrapolated to humans to predict the PK-PD responses of STS, aiming to provide a new theoretical basis for elucidating its mechanism of action and optimizing clinical dosing strategies.

## 2. Materials and Methods

### 2.1. Materials and Reagents

The STS injection (10 mg/2 mL; CAS: 2310302) was purchased from Shanghai Shangyao First Biochemical Pharmaceutical Co., Ltd. (Shanghai, China). The STS standard (purity ≥ 98%; CAS: 69659-80-9) and riboflavin (used as the internal standard (IS); purity ≥ 99%; CAS: 83-88-5) was sourced from Chengdu Lemeitian Pharmaceutical Technology Co., Ltd. (Chengdu, China), and Shanghai Yuanye Biotechnology Co., Ltd. (Shanghai, China) High-performance liquid chromatography (HPLC)-grade methanol and phosphoric acid (CAS: 7664-38-2) were purchased from Tedia High Purity Solvent Co., Ltd. (Anqing, China), and Shanghai Aladdin Biochemical Technology Co., Ltd. (Shanghai, China). Analytical-grade sodium dihydrogen phosphate (AR, CAS: 13472-35-0) was supplied by Shanghai Zhanyun Chemical Co., Ltd. (Shanghai, China). IL-1β and TNF-α ELISA kits (lot no. 202406) were acquired from Jiangsu Enzyme Labeled Biotechnology Co., Ltd. (Wuxi, China). Regenerated cellulose dialysis bags (FDM414-5m) were obtained from Beyotime (Shanghai, China). All other chemicals were of analytical grade.

### 2.2. Experimental Animals

The animal experiment center of the Zhejiang Chinese Medical University supplied specific pathogen-free (SPF)-grade male Sprague-Dawley (SD) rats (weight: 260–300 g, animal license number: SYXK2021-0012). The rats were housed under standard environmental conditions (12 h light/dark cycle, humidity of 45 ± 5%, under 25 ± 2 °C), with free access to standard pelleted laboratory chow and water. The experiment was conducted after a week of adaptive feeding and fasting for 12 h before the experiment. All experimental procedures were strictly in accordance with the Guide for the Care and Use of Laboratory Animals issued by the National Institutes of Health. The experimental method was reviewed and approved by the Animal Ethical and Welfare Committee of Zhejiang Chinese Medical University (Approval Number: IACUC-20221031-25; Approval Date: 31 October 2022).

### 2.3. Construction of Middle Cerebral Artery Occlusion (MCAO)/R Model and Animal Grouping

The MCAO modeling method refers to the previous research of our research group [28]. According to the body weight of rats, atropine (0.04 mg/kg) was injected intramuscularly, and Zoletil 50 (20 mg/kg) was injected intraperitoneally 5 min later. The skin was cut along the midline of the neck, and the right common carotid artery (CCA), external carotid artery (ECA) and internal carotid artery (ICA) were separated. Ligation of CCA and ECA, while using a miniature arterial clip to close ICA. Insert a nylon monofilament wrapped in 0.26 mm polylysine into the right internal carotid artery through the common carotid artery to block the middle cerebral artery (MCA). After inducing ischemia for 1 h, gently pull out the suture and suture the wound. The sham surgery group used the same procedure, but did not insert nylon monofilaments. SD rats were randomly divided into a normal treatment group (NTG), a model treatment group (MTG), a normal control group (NCG), and a model control group (MCG), with 6 rats in each group. The MTG and MCG were used for MCAO modeling, while the NTG and NCG were used for sham surgery. At the same time as ischemia–reperfusion, STS injection (1.68 mL/kg, calculated based on human dose) was immediately administered to the MTG and NTG rats via the tail vein. The NTG and MTG rats were given the same dose (1.68 mL/kg) of physiological saline in the same manner.

### 2.4. HPLC Chromatographic Analysis

#### 2.4.1. Instrument and Chromatographic Conditions

Agilent 1200 HPLC (Hangzhou Zongcan Technology Co., Ltd., Hangzhou, China) and EclipseXDB-C_18_ column (4.6 × 150 mm, 5 μm) were used for chromatographic separation with methanol (A)—0.2% sodium dihydrogen phosphate solution (B) as the mobile phase. The gradient elution process is as follows: 0–15 min, 10-78% A; 15–25 min, 78-78% A; 25–30 min, 78-10% A. The chromatographic conditions were set as follows: injection volume, 10 μL; column temperature, 25 °C; flow rate, 1 mL/min; detection wavelength, 270 nm.

#### 2.4.2. Pretreatment of Plasma Sample

To the plasma sample, add 10 μL of 0.1 mg/mL IS (riboflavin) as the internal standard, followed by the addition of three times the volume of methanol to deproteinize, vortex, and collect the supernatant after centrifugation. A centrifugal concentrator (100-4; Labogene, Allerød, Denmark) was used for centrifugal concentration, and the concentrated samples were stored in a refrigerator at −80 °C for subsequent detection. Before the determination of STS concentration, methanol: a methanol–water mixture (1:1, *v*/*v*) was used to re-dissolve the concentrated sample, and the resolution volume was half of the original sample. The supernatant was obtained after vortexing and centrifugation. The treated plasma samples were then subjected to HPLC analysis for STS determination.

#### 2.4.3. Methodological Validation

The stock reference solutions of STS and IS, with a concentration of 100 μg/mL, were separately prepared in a methanol–water mixture. A series of STS solutions with final concentrations of 0.05, 10, 20, 30, 40, 50, and 60 μg/mL was prepared from the reference solution to verify the linear relationship. To assess the analytical method’s accuracy, precision, stability, and recovery, STS was produced in blank plasma at concentrations of 0.05, 30, and 50 μg/mL. The relative standard deviation (RSD) values of both accuracy and stability were <10%.

### 2.5. PK Study

After drug administration, about 0.5 mL of blood was drawn from the submandibular vein of the MTG and NTG rats at 2, 5, 10, 15, 20, 30, 45, 60, 90, 120, 180, 240, and 360 min into a 1.5 mL heparin sodium centrifuge tube. The plasma was centrifuged at 4500 rpm for 15 min at 4 °C, and the supernatant was collected. A 200 μL volume of plasma was taken for sample pretreatment according to the plasma sample preparation method, and the remaining serum was stored in the −80 °C refrigerator for subsequent PD detection.

### 2.6. PD Study

After injecting physiological saline into the tail vein of the MCG and NCG rats, blood was collected at various time points during the PK study. Then, the plasma sample was centrifuged to recover the supernatant. In accordance with the instructions of the ELISA kit manufacturer, the TNF-α and IL-1β levels in the four groups of plasma samples at different time points were determined by using the INFINITE 200 PRO multifunctional microplate reader (TECAN Austria GmbH, Grödig, Austria).

### 2.7. Plasma Protein-Binding Rate Experiment

The equilibrium dialysis method was employed to determine the plasma protein binding rate (PPB) of STS. Regenerated cellulose dialysis bags were cut into several dialysis bags of approximately 10 cm size; the inside and outside of the dialysis bags were rinsed with ultrapure water, after which the bags were soaked in a balanced dialysate until further use. One end of the dialysis bag was clamped using a special clamp for dialysis bags. After adding 1 mL of blank plasma sample to each of the dialysis bags, the other side was clamped, and the bag was suspended in STS-balanced dialysate with concentrations of 0.1, 0.5, and 1.5 μg/mL. When suspending, it was ensured that the dialysis bags did not stick to the wall of the beaker. The beaker was then placed in a 4 °C refrigerator for balanced dialysis and removed after 48 h. From this, 100 μL of the external dialysate was removed, and 2–3 drops of 10% trichloroacetic acid were added to the dialysate. If the external dialysate became turbid, it was indicative of the plasma protein overflowing from the dialysis bag, which was discarded. Both the plasma and balanced dialysate were collected from each dialysis bag. After sample pretreatment according to the method detailed in Section 2.4.2, HPLC was performed to detect and record the concentration of C_Plasma_ dialysate and C_External fluid_ dialysate, followed by calculation of PPB in accordance with the formula. Three parallel samples were set for each concentration, and the average PPB and RSD of STS at different concentrations were calculated, respectively.

(1) $$\mathrm{PPB}\left(\%\right)=\frac{C_{\mathrm{Plasma}}-C_{\mathrm{External}\;\mathrm{fluid}}}{C_{\mathrm{Plasma}}}\times 100\%$$

In the formula, PPB represents the plasma protein binding rate, C_Plasma_ represents the drug concentration measured in the plasma inside the dialysis bag, and C_External fluid_ represents the drug concentration in the equilibrium dialysate outside the dialysis bag.

### 2.8. PBPK Model Construction

Based on the PBPK model framework in PK Sim software (version 12.1.222, Bayer, Leverkusen, Germany), Python language (version 2024.1) was used to program and construct the model (see the File S1) [29,30]. The PK data measured in the experiment, the physiological characteristics of rats and the physical and chemical parameters related to the drug were introduced. The permeability between plasma and interstitial (p_1_), the permeability between interstitial and cells (p_2_), the permeability between plasma and red blood cells (p_3_), renal clearance rate (C_Lint_K_) and liver clearance rate (C_Lint_L_) were fitted. The following key parameters were obtained. Physiological characteristics of the rats, including their weight, matched organ weight, volume, tissue composition, blood perfusion rates, and other parameters, were generated by the internal module of PK-Sim. Drug-related physicochemical parameters were obtained through the following online databases: DrugBank ADMETlab, and ChEMBL. The p_1_, p_2_, p_3_, C_Lint_K_, and C_Lint_L_ were generated from the model to compare the metabolic differences between the normal and pathological states. The difference between the model and the experimental results was evaluated by the sum of squares of the relative errors (SSREs) using the following calculation formula:

(2) $$\mathrm{SSRE}=\sum \left(\frac{1}{y_{2}}\times {\left(y_{1}-y_{2}\right)}^{2}\right)$$

In the formula, y_1_ represented the predicted value and y_2_ represented the observed value. The index can better reflect the relative deviation between the predicted value and the actual observation value by standardizing the error term.

In order to extrapolate the validated rat PBPK model to the human body, human physiological parameters were integrated into the model while retaining the basic structure of the model. For the key PK parameters C_Lint_K_ and C_Lint_L_, the interspecies extrapolation was performed according to the allometric growth scaling method [17] using the following calculation formula:

(3) $${\mathrm{CL}}_{\mathrm{human}}={\mathrm{CL}}_{\mathrm{animal}}\times {\left(\frac{W_{\mathrm{human}}}{W_{\mathrm{animal}}}\right)}^{0.75}$$

In the formula, CL_human_ represents the predicted human drug clearance, CL_animal_ represents the drug clearance in the experimental animal, W_human_ represents the standard human body weight, taken as 70 kg, and W_animal_ represents the average body weight of the experimental rats. The allometric scaling corrects ‘species difference’ rather than ‘pathological state difference’. In this study, rat PBPK model results showed a significant decrease in renal and liver clearance caused by cerebral ischemia–reperfusion injury, in which the clearance rate of normal rats and model rats has included pathological factors. In recent years, in the field of PBPK modeling, various PBPK studies have also used the 0.75 power allometric scaling method to infer the clearance rate between species [31,32]. Finally, the scaled human clearance parameters and human physiological parameters were combined to build a human PBPK model.

### 2.9. PBPK-PD Model Construction

The PBPK model of rats detailed in Section 2.7 was combined with the PD results in Section 2.5 to construct the PBPK-PD model. The following formula was used to describe the model:

(4) $$\frac{d{\tilde{C}}_{1}}{\mathrm{dt}}=\;k_{1}\left(C_{\mathrm{venous}\;\mathrm{plasma}}-{\tilde{C}}_{1}\right)$$

(5) $$E=\frac{{{\tilde{C}}_{1}}^{r_{1}}}{{E_{50}}^{r_{1}}+{{\tilde{C}}_{1}}^{r_{1}}}$$

In the formulas, $\frac{d{\tilde{C}}_{1}}{dt}$ represents the derivative of effect-site concentration with respect to time, which is the rate of change, ${\tilde{C}}_{1}$ represents the concentration of the effect compartment, k_1_ represents the first-order rate constant of the effect compartment, C_venous plasma_ represents the concentration of venous plasma, r_1_ reflects the steepness of the effect curve and E_50_ has been defined as the drug concentration that produces half of the maximal effect.

### 2.10. Data Analysis

GraphPad 8.0.2 software was used for mapping and statistical analysis, and a two tailed *p* value less than 0.05 was considered to indicate a statistically significant. The mean ± standard deviation ($\bar{x}$ ± s) is used to express data.

## 3. Results

### 3.1. Methodological Validation

**Figure 2:**
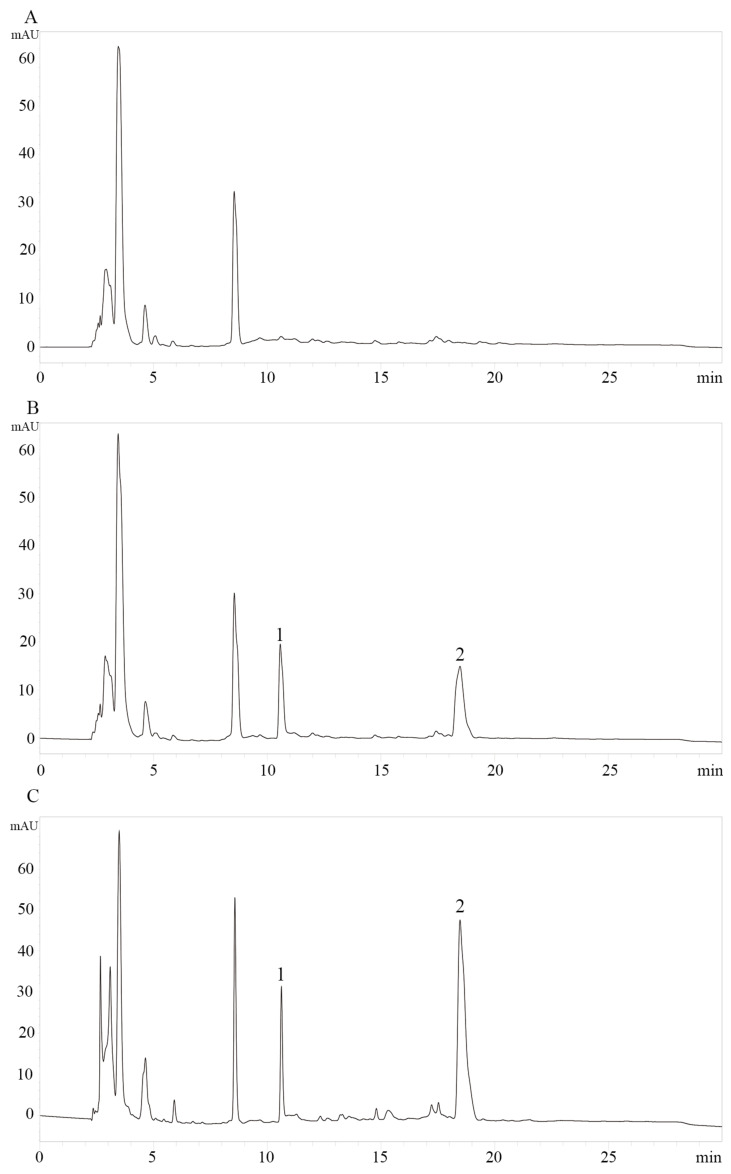
Typical chromatogram of rat plasma (270 nm). (**A**) Blank plasma. (**B**) Blank plasma + IS + STS; (**C**) 5 min after administration, plasma sample + IS; 1: IS; 2: STS.

A sensitive and accurate HPLC technique was established for determining the concentration of STS in rat plasma. The determination of STS was not interfered with by endogenous substances or IS, which confirmed good specificity (Figure 2). Excellent linearity was found over the entire concentration range studied (y = 207.58x + 0.0117, R^2^ = 0.9997). The lowest detection limit for STS was 0.03 μg/mL. Both intra-day and inter-day precisions satisfied the acceptance criteria with RSD being less than 10% (Table 1). The method also showed satisfactory recovery (Table 1). Stability studies confirmed that STS remained stable in plasma under various storage conditions, including 4 °C for 24 h, three freeze–thaw cycles at −80 °C, and storage at −80 °C for 2 weeks (Table 1).

In summary, the validated HPLC method meets the technical requirements for bioanalytical applications and is suitable for subsequent PK studies.

### 3.2. Pharmacokinetics

**Figure 3:**
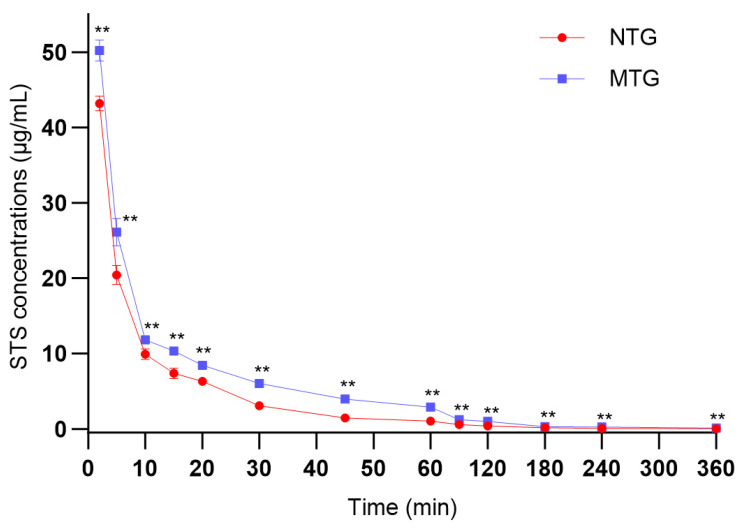
Concentration–time curves of STS in NTG and MTG rats ($\overset{-}{x}$ ± s, *n* = 6). Compared with NTG: ** *p* < 0.01.

The plasma concentration–time profiles of STS in rats are shown in Figure 3. Following tail vein injection, STS rapidly reached peak concentration and then declined over time. At every time point, plasma STS concentrations in the MTG were significantly higher than those in the NTG (*p* < 0.01).

### 3.3. Pharmacology

**Figure 4:**
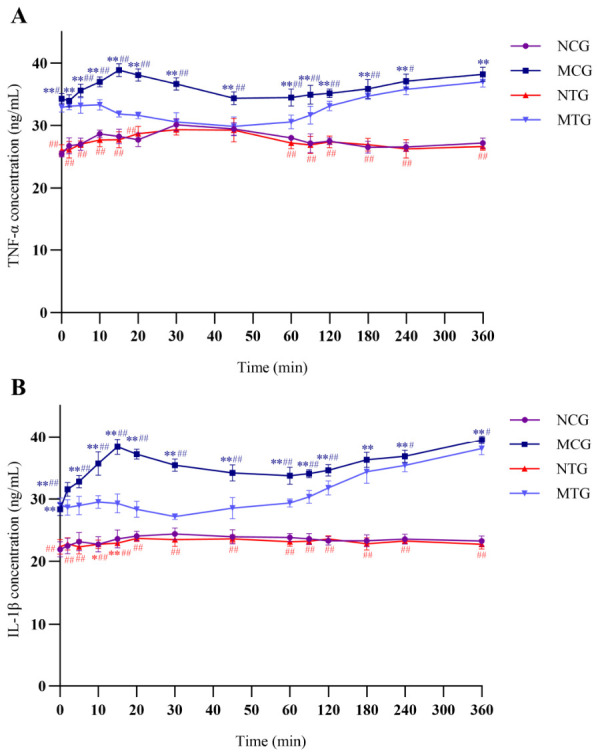
Concentration–time curves of TNF-α and IL-1β in serum/plasma of NCG, MCG, NTG, and MTG rats ($\overset{-}{x}$ ± s, *n* = 6). (**A**) TNF-α; (**B**) IL-1β. Compared with NCG rats: ** *p* < 0.01; * *p* < 0.05. Compared with MTG rats: ^##^ *p* < 0.01; ^#^ *p* < 0.05.

Inflammatory markers in peripheral plasma were measured in the NCG, MCG, NTG, and MTG at multiple time points, and corresponding concentration–time curves were constructed (Figure 4). The results showed that inflammatory levels in the MCG were significantly higher than those in the NCG (*p* < 0.01), and those in the MTG were significantly higher than in the NTG (*p* < 0.01). PD responses in the NTG and MTG were statistically significant at most time points (*p* < 0.05). Compared with the MCG, the MTG exhibited lower overall inflammatory levels at most time points, with statistical significance (*p* < 0.05). In contrast, IL-1β and TNF-α levels in the NTG and NCG remained relatively stable, with no marked fluctuations.

### 3.4. Plasma Protein Binding Rate (PPB)

The PPB of STS was evaluated in normal and model rats at low, medium, and high concentrations (0.1, 0.5, and 1.5 μg/mL). The results showed that the PPB rates of STS in both normal and model rats exceeded 90% (Table 2).

### 3.5. PBPK Model

**Figure 5:**
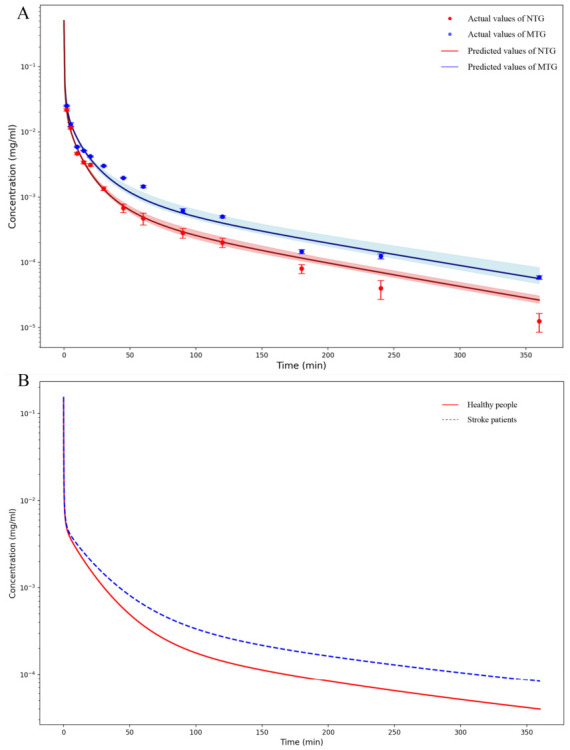
Physiologically based pharmacokinetic (PBPK) curves ($\overset{-}{x}$ ± s, *n* = 6). (**A**) NTG and MTG rats. (**B**) Healthy subjects and stroke patients.

Based on the physicochemical properties of STS, rat physiological parameters, and PK data, a rat PBPK model was successfully established (Figure 5). The key fitted parameters are summarized in Table 3.

Analysis of the fitted parameters showed no statistically significant differences in the permeability coefficients (p_1_, p_2_, and p_3_), whereas the C_Lint_K_ and C_Lint_L_ were significantly reduced (*p* < 0.05). These results indicate that CIRI mainly changes STS clearance, rather than tissue distribution.

Based on the rat-derived C_Lint_K_ and C_Lint_L_ values, corresponding human clearance parameters were estimated using allometric scaling (Table 3). By integrating the rat PBPK model structure with human physiological parameters and the extrapolated clearance values, a human PBPK model was constructed, and key PK parameters were obtained through model fitting (Table 3). The simulated concentration–time profiles indicate that STS undergoes rapid distribution and elimination in humans, with slower elimination under pathological conditions compared with healthy individuals (Figure 5).

### 3.6. PBPK-PD Model Establishment

**Figure 6:**
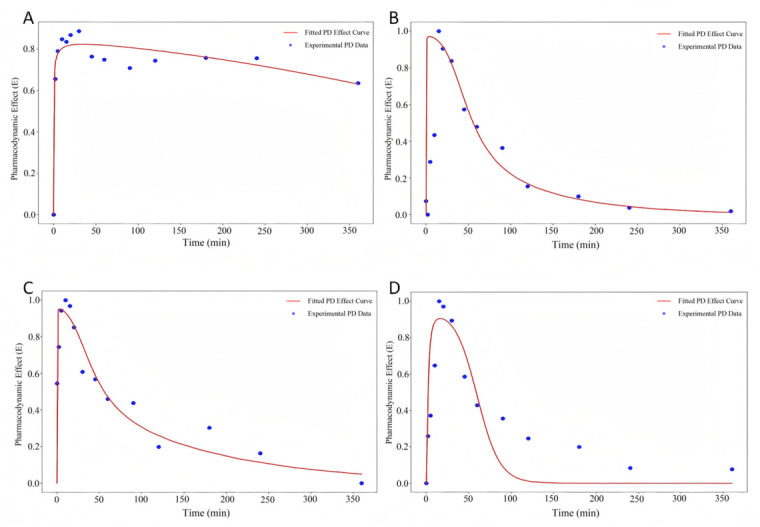
PBPK-PD curves of STS predicted efficacy results and actual efficacy results in NTG and MTG rats. (**A**) TNF-α in NTG; (**B**) TNF-α in MTG; (**C**) IL-1β in NTG; (**D**) IL-1β in MTG.

To better characterize the anti-inflammatory effects of STS, the absolute differences in TNF-α and IL-1β levels between treatment and corresponding control groups at each time point were used as PD endpoints. These endpoints were integrated with the PBPK model described in Section 3.5 to develop a PBPK-PD model, with parameter estimates summarized in Table 4. The time–effect relationships between predicted and observed effects of STS in the NTG and MTG are shown in Figure 6.

## 4. Discussion

The pathogenesis of CIRI is multifactorial and complex. Neuroinflammation plays a pivotal role in the progression of brain injury, and stroke severity is closely associated with the magnitude of the inflammatory response [33]. Many traditional Chinese medicines alleviate CIRI by postischemic inflammation [34]. STS, a major bioactive derivative of *S. miltiorrhiza* (Danshen), exhibits improved bioavailability and demonstrates anti-inflammatory and cardiovascular protective effects in clinical settings [20]. However, existing studies are largely limited to conventional PK analyses of intravenous STS in rodents [35,36], with no reports addressing PBPK-PD modeling or comparative metabolic behavior between normal and CIRI conditions. In this context, the present study establishes, for the first time, PBPK-PD models of STS in both normal and CIRI rats, providing quantitative support for its rational clinical application.

The PK results indicate that systemic exposure to STS in the MTG is significantly higher than that in the NTG. This suggests that CIRI reduces the clearance of STS, resulting in a prolonged apparent half-life and increased systemic retention. However, the overall elimination of STS in both normal and diseased rats is rapid, indicating fast distribution and metabolism, consistent with previous PK results in normal rats [37]. PBPK model fitting further demonstrates that, compared with the NTG, tissue permeability parameters of STS in the MTG remain unchanged, whereas both renal and hepatic clearance are significantly reduced. This result may be due to the systemic inflammatory response downregulation of metabolic enzymes and transporters in the liver and kidney. Research has shown that pro-inflammatory cytokines can significantly inhibit the expression and activity of cytochrome P450 enzymes while downregulating the expression of drug transporters such as organic anion transporters [38,39,40]. In contrast, permeability parameters describe the passive diffusion process of small molecule drugs between plasma, interstitial fluid, and cells, mainly determined by the physicochemical properties of the drug itself, including molecular weight, lipophilicity, and hydrogen bonding ability, rather than relying on the expression or function of active transporters [41]. Therefore, they are less affected by validation. In summary, the decrease in drug clearance rate under systemic inflammation is mainly caused by the inhibition of metabolic enzymes and transporters. These findings suggest that CIRI impairs systemic clearance mainly by affecting liver and kidney function rather than changing tissue distribution. Given that STS predominantly accumulates in and is cleared by the liver and kidneys [36], the observed reduction in clearance may be attributable to CIRI-induced systemic inflammation and multiorgan dysfunction. Stroke destroys cellular homeostasis. In addition to directly damaging the brain, inflammation can also spread through the blood, destroy the homeostasis of distant organs, and damage cell functions such as cell membranes, proteins, and nucleic acids [42]. In parallel, systemic inflammation, oxidative stress, and activation of the sympathetic nervous system contribute to acute kidney injury and hepatocellular damage [43]. CIRI significantly enhances inflammatory responses in the liver and kidneys, leading to organ injury [44,45].

Building on the successful development and validation of the rat PBPK model, the present study further extrapolated the model to humans to quantitatively predict the PK behavior of STS and to assess changes in systemic exposure under pathological conditions. The model prediction results show that STS has an extremely short half-life in humans, consistent with rapid attainment of peak plasma concentrations following intravenous administration and subsequent rapid distribution and clearance. Among them, because the PBPK model of this study regards the vein as a whole, the predicted T_max_ after intravenous injection is 0. But in reality, because it is divided into different veins, the actual T_max_ is not 0. These results imply that treatment with only one intravenous bolus dose might fail to sustain effective drug concentrations over time. It is thus reasonable to consider appropriate adjustments to dosing regimens in clinical settings, so as to keep plasma drug levels within the therapeutic window. Alternative delivery strategies, such as switching from a one-off intravenous bolus to continuous infusion or adopting multiple divided doses, can help maintain sufficient drug exposure at the target site.

Compared with the normal condition, STS exposure under pathological conditions increased significantly (by approximately 37%), which indicates that CIRI-induced systemic pathophysiological changes significantly affect the metabolic process of STS, potentially through alterations in blood flow after stroke. CIRI can increase blood viscosity and reduce erythrocyte deformability, thereby affecting drug distribution and clearance [46]. In addition, ischemia–reperfusion may impair the function of drug transporters, further altering tissue distribution and elimination [47]. Disruption of BBB integrity may also enhance drug penetration into the central nervous system and modify overall PK behavior [48]. These findings suggest that the impact of the patient’s pathological status on STS PK must be carefully considered. In clinical practice, direct application of dosing regimens derived from healthy populations may result in higher-than-expected drug exposure in patients with stroke, thereby increasing the potential safety risks. Conversely, increased exposure may enhance therapeutic efficacy. Our PD results demonstrate that STS significantly reduces pro-inflammatory cytokine levels, with effects positively correlated with plasma concentrations. Therefore, elevated exposure under pathological conditions may confer enhanced anti-inflammatory and neuroprotective effects. From a clinical perspective, an initial dose reduction may be warranted to mitigate potential toxicity. As the pathological condition improves, dosing regimens should be adjusted accordingly to achieve individualized therapy, balancing efficacy and safety.

The physiological parameters of the human body vary depending on age, gender, and ethnicity. Therefore, we can adjust the relevant physiological parameters through the PBPK model, predict the pharmacokinetic characteristics of patients under different physiological conditions, and provide medication guidance for different populations.

Microglia are key cellular sensors that trigger neuroinflammatory responses after ischemic injury. Ischemia–reperfusion injury can trigger microglial activation, prompting the secretion of proinflammatory mediators such as TNF-α, IL-6, and IL-1β [49]; these mediators in turn exacerbate neuronal necrosis, BBB disruption and cerebral edema progression [50]. Meanwhile, inflammatory stimuli can activate nuclear factor-κB (NF-κB), which migrates into the nucleus to modulate downstream inflammatory signaling cascades. This process drives the synthesis and secretion of more cytokines, eventually forming a self-amplifying positive feedback loop [51]. In the present work, TNF-α and IL-1β were adopted as PD biomarkers to mirror the evolving process of CIRI in rat models, as well as to contrast the PD changes between normal rats and MCAO model rats after intravenous administration of STS.

Levels of TNF-α and IL-1β in the NCGs and NTGs fluctuated slightly and stayed at a steady level throughout the observation period. When compared with the MCGs, the two PD indicators in the MTG remained at distinctly lower levels all the time. Statistical analysis revealed obvious differences in these two indicators between the NCG and MCG, NTG and MTG, as well as the MCG and MTG (*p* < 0.05). These results suggest that the therapeutic efficacy of STS against CIRI is largely achieved by modulating inflammatory reactions. After ischemic injury, STS intervention can relieve BBB damage and restrict peripheral inflammatory cells from infiltrating brain tissues, which in turn mitigates neuroinflammatory responses and facilitates functional recovery [52]. STS is capable of restraining NF-κB nuclear translocation and lowering the expression of TNF-α and IL-1β [53]. Existing evidence also demonstrates that STS relieves ischemic brain damage by blocking overactivated inflammatory signaling pathways [54], which aligns well with the results obtained in this investigation. Research has found that CIRI not only causes focal brain damage, but also triggers cytokine storms through systemic inflammatory responses [55]. Cytokine storm is caused by excessive activation of immune cells, leading to the release of cytokines in large quantities, resulting in dysregulation of inflammatory responses and self-reinforcing feedback, ultimately endangering life [56]. Cytokine storm can cause multiple organ dysfunction, including acute kidney injury, liver injury, and even death [57,58,59]. This mechanism also explains the liver and kidney function damage under CIRI status in this study, which directly led to a decrease in clearance ability.

The present work assessed the PK-PD correlation of STS by combining dynamic changes in plasma drug levels and corresponding PD responses over time. The parameter R_1_ was adopted to evaluate drug sensitivity across the effective concentration range and characterizes the gradient feature of the exposure–response curve, while E_50_ served as an indicator for evaluating drug safety performance. Model calculation results revealed that the R1 values of TNF-α and IL-1β derived from the established PBPK-PD model were higher than 1 in the MTG rats. Around the median effective concentration ED_50_, the anti-inflammatory efficacy of STS varied considerably. In addition, the time required to reach the maximum PD effect occurred later than the peak time of plasma drug concentration, demonstrating an obvious hysteresis in the pharmacological response. Collectively, the PBPK-PD model constructed based on MCAO rat data offers a quantitative analytical framework for rational dosing design. It also lays a methodological foundation to advance the clinical transformation and application of STS treatment in human trials.

This study confirmed that STS can significantly inhibit the inflammatory response in rats, thereby reducing the pathological damage caused by CIRI. On the basis of the established rat model, a human PBPK model was further constructed to characterize the in vivo disposition of STS among healthy populations and stroke patients alike. This work mainly centered on exploring the PK-PD correlation of STS, while the intrinsic molecular mechanisms responsible for its pharmacological action were not systematically clarified. Subsequent research can be combined with behavioral studies to better evaluate the therapeutic effect of STS, as well as to detect the distribution of STS in rats over time and explore the molecular mechanisms of STS anti-inflammatory effects, in order to optimize and improve the model and enhance its predictive accuracy for clinical drug administration. In addition, based on the PBPK model developed in this study, the model parameters can be adjusted according to the severity of stroke in the future to simulate the metabolic process of STS in the human body under different pathological states, evaluate the optimal dosage, and continuously optimize and improve the model according to clinical results, so that it can be better applied in clinical drug administration. In addition, this study found a decrease in clearance rate after stroke, which may become one of the indicators for evaluating the degree of recovery after stroke. In subsequent studies, the clearance rate of stroke patients with different degrees of severity can be detected to verify whether this indicator can be used as a standard for rehabilitation assessment.

## 5. Conclusions

In this study, the PK-PD characteristics of STS in normal and CIRI rat models were systematically characterized. Plasma STS levels were adopted as PK endpoints to delineate concentration–time profiles and PPB, which furnished fundamental data support for the construction of PBPK models. TNF-αand IL-1β were chosen as typical PD biomarkers for assessing the anti-inflammatory effects exerted by STS. Using the above experimental data, we successfully constructed PBPK-PD models for STS delivered via intravenous (tail vein) injection in both normal and MCAO model rats. Even so, the present investigation was conducted exclusively in rat models, a limitation that hinders its straightforward translation into clinical practice. Subsequent research can expand the current modeling framework to more animal species and eventually translate it into clinical trials, with the aim of verifying its predictive reliability, safety and therapeutic potency. Such efforts will lay an experimental foundation for refining dosing strategies and elevating the clinical efficacy of STS in the treatment of ischemic stroke.

## Figures and Tables

**Table 1:** Precision, recovery and stability of STS in rat plasma ($\overset{-}{x}$ ± s, *n* = 6).

Concentration (µg/mL)	Precision RSD (%)		Stability RSD (%)			Recovery Rate (%)
	Intra Day	Inter Day	Store at 4 °C for 24 h	Freeze–Thaw Stability	Store at −80 °C for Two Weeks	
0.5	4.73	3.70	6.06	8.16	5.59	97.16 ± 3.56
30	3.29	5.86	5.78	5.19	4.11	97.76 ± 2.18
50	3.46	4.52	4.46	5.22	3.54	102.78 ± 1.65

**Table 2:** Plasma protein binding rates of rats with different concentrations of STS ($\overset{-}{x}$ ± s, *n* = 6).

Concentrations (µg/mL)	NTG		MTG	
	Average PPB at Different Concentrations (%, *n* = 6)	Average PPB (%)	Average PPB at Different Concentrations (%, *n* = 6)	Average PPB (%)
0.5	91.66 ± 4.78	93.85 ± 4.00	94.35 ± 2.32	95.94 ± 1.83
30	92.75 ± 2.97		96.86 ± 0.81	
50	97.15 ± 1.14		96.62 ± 0.71	

**Table 3:** Key pharmacokinetic parameters of STS in NTG/MTG rats, healthy subjects, and stroke patients estimated by PBPK model (mean ± s, *n* = 6). Comparison with NTG: ** *p* < 0.01; * *p* < 0.05.

PK Parameters	NTG	MTG	Healthy People	Stroke Patients
p1	0.0037 ± 0.00066	0.0036 ± 0.0012	/	/
p2	1.90 × 10^−5^ ± 2.17 × 10^−6^	2.02 × 10^−5^ ± 2.46 × 10^−6^	/	/
p3	0.00022 ± 0.00013	0.00023 ± 0.00020	/	/
C_Lint_K_	11.25 ± 1.47	7.61 ± 0.87 **	144.712	80.699
C_Lint_L_	3.22 ± 1.22	1.79 ± 0.83 *	506.272	342.394
C_max_ (μg/mL)	/	/	154	154
T_max_ (min)	/	/	0.000	0.000
t_1/2_ (min)	/	/	0.0511	0.0509
AUC_(0-t)_ (min/mL)	/	/	0.139	0.191

**Table 4:** PBPK-PD model equation.

Index	Group	NTG	SSR	R^2^
TNF-α	NTG	$\frac{d{\tilde{C}}_{1}}{\mathrm{dt}}=0.007560\left(C_{\mathrm{venous}\;\mathrm{plasma}}-{\tilde{C}}_{1}\right)$ $E=\frac{{{\tilde{C}}_{1}}^{0.483367}}{0.000461^{0.483367}+{{\tilde{C}}_{1}}^{0.483367}}$	0.041665	0.932863
	MTG	$\frac{d{\tilde{C}}_{1}}{\mathrm{dt}}=0.018579\left(C_{\mathrm{venous}\;\mathrm{plasma}}-{\tilde{C}}_{1}\right)$ $E=\frac{{{\tilde{C}}_{1}}^{6.654087}}{0.016238^{6.654087}+{{\tilde{C}}_{1}}^{6.654087}}$	0.220268	0.857094
IL-1β	NTG	$\frac{d{\tilde{C}}_{1}}{\mathrm{dt}}=0.144272\left(C_{\mathrm{venous}\;\mathrm{plasma}}-{\tilde{C}}_{1}\right)$ $E=\frac{{{\tilde{C}}_{1}}^{0.889158}}{{\;0.006758}^{0.889158}+{{\tilde{C}}_{1}}^{0.889158}}$	0.433137	0.672019
	MTG	$\frac{d{\tilde{C}}_{1}}{\mathrm{dt}}=0.022005\left(C_{\mathrm{venous}\;\mathrm{plasma}}-{\tilde{C}}_{1}\right)$ $E=\frac{{{\tilde{C}}_{1}}^{4.503805}}{0.016438^{4.503805}+{{\tilde{C}}_{1}}^{4.503805}}$	0.262418	0.820067

## Data Availability

Data will be made available on request.

## Supplementary Materials

The following supporting information can be downloaded at: https://www.mdpi.com/article/10.3390/biology15110827/s1. File S1: PBPK model equations.

## Abbreviations

CIRI	Cerebral ischemia–reperfusion injury
ELISA	Enzyme-linked immunosorbent assay
HPLC	High-performance liquid chromatography
IL-1β	Interleukin-1β
IS	Internal standard
LLOD	Lower limit of detection
MCAO	Middle cerebral artery occlusion
MCG	Model control group
MTG	Model treatment group
NCG	Normal control group
NTG	Normal treatment group
RSD	Relative standard deviation
R^2^	The square of the correlation coefficient
TNF-α	Tumor necrosis factor-α
STS	Sodium tanshinone IIA sulfonate
