# Supplementary material for: Physiologically Based Pharmacokinetic–Pharmacodynamic-Based Quantification of Exposure–Response for Sodium Tanshinone IIA Sulfonate in Normal and Cerebral Ischemia–Reperfusion Injury Rats
Source: Biology (Basel). 2026 May 24;15(11):827. doi: 10.3390/biology15110827 (PMC13255987; doi:10.3390/biology15110827)
Supplement: Supplementary file 1 [file biology-15-00827-s001.zip › File S1.pdf]

Assumption of compartments: The body is divided into 15 main organs: liver, kidney, stomach, small intestine, large intestine, gonads, spleen, lung, bone, brain, fat, muscle, skin, pancreas, and heart, as well as two blood compartments: venous blood and arterial blood. Each organ is further divided into four compartments: red blood cells, plasma, intercellular matrix, and cells. The blood compartment is divided into two compartments: red blood cells and plasma. The distribution and transport process of compounds between different compartments in the body are described by permeability and partition coefficient. Consider each compartment as fully mixed.

The parameters are defined on the last page.

Lumen:

$$K_a = \frac{PS_{lumen}}{V_{lumen}}$$

$$\frac{dX_{lumen}}{dt} = -K_a X_{lumen}$$

Stomach(ug/h):

Plasma layer: $\frac{dX_{st}}{dt} = Q_{st} \times \frac{X_{art}}{V_{art}} - Q_{st} \times \frac{X_{st}}{V_{st}} + (\frac{X'_{st} \times PS_{st1}}{V'_{st} \times K'_{st}} - PS_{st1} \times \frac{X_{st}}{V_{st}} - \frac{PS_{st3} \times X_{st}}{V_{st}} + \frac{X'''_{st} \times PS_{st3}}{V'''_{st} \times K'''_{st}}) \times f_u$

Intercellular stromal layer: $\frac{dX'_{st}}{dt} = (\frac{PS_{st1} \times X_{st}}{V_{st}} - \frac{X'_{st} \times PS_{st1}}{V'_{st} \times K'_{st}} - \frac{PS_{st2} \times X'_{st}}{V'_{st} \times K'_{st}} + \frac{X''_{st} \times PS_{st2}}{V''_{st} \times K''_{st}}) \times f_u$

Cell layer: $\frac{dX''_{st}}{dt} = (\frac{PS_{st2} \times X'_{st}}{V'_{st} \times K'_{st}} - \frac{X''_{st} \times PS_{st2}}{V''_{st} \times K''_{st}}) \times f_u$

Erythrocyte layer: $\frac{dX'''_{st}}{dt} = (\frac{PS_{st3} \times X_{st}}{V_{st}} - \frac{X'''_{st} \times PS_{st3}}{V'''_{st} \times K'''_{st}}) \times f_u$

Small intestine:

$$\frac{dX_{sm}}{dt} = Q_{sm} \times \frac{X_{art}}{V_{art}} - Q_{sm} \times \frac{X_{sm}}{V_{sm}} + (\frac{X'_{sm} \times PS_{sm1}}{V'_{sm} \times K'_{sm}} - PS_{sm1} \times \frac{X_{st}}{V_{st}} - \frac{PS_{sm3} \times X_{sm}}{V_{sm} \times K_m} + \frac{X'''_{sm} \times PS_{sm3}}{V'''_{sm} \times K'''_{sm}}) \times f_u + K_a X_{lumen}$$

Intercellular stromal layer: $\frac{dX'_{sm}}{dt} = (\frac{PS_{sm1} \times X_{st}}{V_{sm}} - \frac{X'_{sm} \times PS_{sm1}}{V'_{sm} \times K'_{sm}} - \frac{PS_{sm2} \times X'_{sm}}{V'_{sm} \times K'_{sm}} + \frac{X''_{sm} \times PS_{sm2}}{V''_{sm} \times K''_{sm}}) \times f_u$

Cell layer: $\frac{dX''_{sm}}{dt} = (\frac{PS_{sm2} \times X'_{sm}}{V'_{sm} \times K'_{sm}} - \frac{X''_{sm} \times PS_{sm2}}{V''_{sm} \times K''_{sm}}) \times f_u$

Erythrocyte layer: $\frac{dX'''_{sm}}{dt} = (\frac{PS_{sm3} \times X_{sm}}{V_{sm}} - \frac{X'''_{sm} \times PS_{sm3}}{V'''_{sm} \times K'''_{sm}}) \times f_u$

Large intestine:

$$\frac{dX_{la}}{dt}=Q_{la}\times\frac{X_{art}}{V_{art}}-Q_{la}\times\frac{X_{la}}{V_{la}}+(\frac{X_{la}^{'}\times PS_{la1}}{V_{la}^{'}\times K_{la}^{'}}-PS_{la1}\times\frac{X_{la}}{V_{la}}-\frac{PS_{la3}\times X_{la}}{V_{la}\times K_{m}}+\frac{X_{la}^{'''}\times PS_{la3}}{V_{la}^{'''}\times K_{la}^{'''}})\times f_{u}$$

Intercellular stromal layer: $\frac{dX_{la}^{'}}{dt}=(\frac{PS_{la1}\times X_{la}}{V_{la}}-\frac{X_{la}^{'}\times PS_{la1}}{V_{la}^{'}\times K_{la}^{'}}-\frac{PS_{la2}\times X_{la}^{'}}{V_{la}^{'}\times K_{la}^{'}}+\frac{X_{la}^{''}\times PS_{la2}}{V_{la}^{''}\times K_{la}^{''}})\times f_{u}$

Cell layer: $\frac{dX_{la}^{''}}{dt}=(\frac{PS_{la2}\times X_{la}^{'}}{V_{la}^{'}\times K_{la}^{'}}-\frac{X_{la}^{''}\times PS_{la2}}{V_{la}^{''}\times K_{la}^{''}})\times f_{u}$

Erythrocyte layer: $\frac{dX_{la}^{'''}}{dt}=(\frac{PS_{la3}\times X_{la}}{V_{la}}-\frac{X_{la}^{'''}\times PS_{la3}}{V_{la}^{'''}\times K_{la}^{'''}})\times f_{u}$

Pancreas:

$$\frac{dX_{pa}}{dt}=Q_{pa}\times\frac{X_{art}}{V_{art}}-Q_{pa}\times\frac{X_{pa}}{V_{pa}}+(\frac{X_{pa}^{'}\times PS_{pa1}}{V_{pa}^{'}\times K_{pa}^{'}}-PS_{pa1}\times\frac{X_{pa}}{V_{pa}}-\frac{PS_{pa3}\times X_{pa}}{V_{pa}}+\frac{X_{pa}^{'''}\times PS_{pa3}}{V_{pa}^{'''}\times K_{pa}^{'''}})\times f_{u}$$

Intercellular stromal layer: $\frac{dX_{pa}^{'}}{dt}=(\frac{PS_{pa1}\times X_{pa}}{V_{pa}}-\frac{X_{pa}^{'}\times PS_{pa1}}{V_{pa}^{'}\times K_{pa}^{'}}-\frac{PS_{pa2}\times X_{pa}^{'}}{V_{pa}^{'}\times K_{pa}^{'}}+\frac{X_{pa}^{''}\times PS_{pa2}}{V_{pa}^{''}\times K_{pa}^{''}})\times f_{u}$

Cell layer: $\frac{dX_{pa}^{''}}{dt}=(\frac{PS_{pa2}\times X_{pa}^{'}}{V_{pa}^{'}\times K_{pa}^{'}}-\frac{X_{pa}^{''}\times PS_{pa2}}{V_{pa}^{''}\times K_{pa}^{''}})\times f_{u}$

Erythrocyte layer: $\frac{dX_{pa}^{'''}}{dt}=(\frac{PS_{pa3}\times X_{pa}}{V_{pa}}-\frac{X_{pa}^{'''}\times PS_{pa3}}{V_{pa}^{'''}\times K_{pa}^{'''}})\times f_{u}$

Spleen:

$$\frac{dX_{sp}}{dt}=Q_{sp}\times\frac{X_{art}}{V_{art}}-Q_{sp}\times\frac{X_{sp}}{V_{sp}}+(\frac{X_{sp}^{'}\times PS_{sp1}}{V_{sp}^{'}\times K_{sp}^{'}}-PS_{sp1}\times\frac{X_{sp}}{V_{sp}}-\frac{PS_{sp3}\times X_{sp}}{V_{sp}}+\frac{X_{sp}^{'''}\times PS_{sp3}}{V_{sp}^{'''}\times K_{sp}^{'''}})\times f_{u}$$

Intercellular stromal layer: $\frac{dX_{sp}^{'}}{dt}=(\frac{PS_{sp1}\times X_{sp}}{V_{pa}}-\frac{X_{sp}^{'}\times PS_{sp1}}{V_{sp}^{'}\times K_{sp}^{'}}-\frac{PS_{sp2}\times X_{sp}^{'}}{V_{sp}^{'}\times K_{sp}^{'}}+\frac{X_{sp}^{''}\times PS_{sp2}}{V_{sp}^{''}\times K_{sp}^{''}})\times f_{u}$

Cell layer: $\frac{dX_{sp}^{''}}{dt}=(\frac{PS_{sp2}\times X_{sp}^{'}}{V_{sp}^{'}\times K_{sp}^{'}}-\frac{X_{sp}^{''}\times PS_{sp2}}{V_{sp}^{''}\times K_{sp}^{''}})\times f_{u}$

Erythrocyte layer: $\frac{dX_{sp}^{'''}}{dt}=(\frac{PS_{sp3}\times X_{sp}}{V_{sp}}-\frac{X_{sp}^{'''}\times PS_{sp3}}{V_{sp}^{'''}\times K_{sp}^{'''}})\times f_{u}$

Portal vein:

$$Q_{po}=Q_{sp}+Q_{pa}+Q_{la}+Q_{sm}+Q_{st}$$

Liver:

$$\frac{dX_{li}}{dt}=Q_{li}\times\frac{X_{art}}{V_{art}}-(Q_{li}+Q_{po})\times\frac{X_{li}}{V_{li}}+Q_{sp}\times\frac{X_{sp}}{V_{sp}}+Q_{pa}\times\frac{X_{pa}}{V_{pa}}+Q_{la}\times\frac{X_{la}}{V_{la}}+Q_{sm}\times\frac{X_{sm}}{V_{sm}}+Q_{st}\times\frac{X_{st}}{V_{st}}+$$
$$(\frac{X'_{li}\times PS_{li1}}{V'_{li}\times K'_{li}}-\frac{PS_{li1}\times X_{li}}{V_{li}}-\frac{PS_{li3}\times X_{li}}{V_{li}}+\frac{X'''_{li}\times PS_{li3}}{V'''_{li}\times K'''_{li}})\times f_u-C_{Lint\_L}\frac{X_{li}}{V_{li}}\times f_u$$

Intercellular stromal layer: $\frac{dX'_{li}}{dt}=(\frac{PS_{li1}\times X_{li}}{V_{li}}-\frac{X'_{li}\times PS_{li1}}{V'_{li}\times K'_{li}}-\frac{PS_{li2}\times X'_{li}}{V'_{li}\times K'_{li}}+\frac{X''_{li}\times PS_{li2}}{V''_{li}\times K''_{li}})\times f_u$

Cell layer: $\frac{dX''_{li}}{dt}=(\frac{PS_{li2}\times X'_{li}}{V'_{li}\times K'_{li}}-\frac{X''_{li}\times PS_{li2}}{V''_{li}\times K''_{li}})\times f_u$

Erythrocyte layer: $\frac{dX'''_{li}}{dt}=(\frac{PS_{li3}\times X_{li}}{V_{li}}-\frac{X'''_{li}\times PS_{li3}}{V'''_{li}\times K'''_{li}})\times f_u$

Kidney:

$$\frac{dX_{kid}}{dt}=Q_{kid}\times\frac{X_{art}}{V_{art}}-Q_{kid}\times\frac{X_{kid}}{V_{kid}}+(\frac{X'_{kid}\times PS_{kid1}}{V'_{kid}\times K'_{kid}}-\frac{PS_{kid1}\times X_{kid}}{V_{kid}}-\frac{PS_{kid3}\times X_{kid}}{V_{kid}}+\frac{X'''_{kid}\times PS_{kid3}}{V'''_{kid}\times K'''_{kid}}-C_{Lint\_K}\frac{X_{kid}}{V_{kid}})\times f_u$$

Intercellular stromal layer: $\frac{dX'_{kid}}{dt}=(\frac{PS_{kid1}\times X_{kid}}{V_{kid}}-\frac{X'_{kid}\times PS_{kid1}}{V'_{kid}\times K'_{kid}}-\frac{PS_{kid2}\times X'_{kid}}{V'_{kid}\times K'_{kid}}+\frac{X''_{kid}\times PS_{kid2}}{V''_{kid}\times K''_{kid}})\times f_u$

Cell layer: $\frac{dX''_{kid}}{dt}=(\frac{PS_{kid2}\times X'_{kid}}{V'_{kid}\times K'_{kid}}-\frac{X''_{kid}\times PS_{kid2}}{V''_{kid}\times K''_{kid}})\times f_u$

Erythrocyte layer: $\frac{dX'''_{kid}}{dt}=(\frac{PS_{kid3}\times X_{kid}}{V_{kid}}-\frac{X'''_{kid}\times PS_{kid3}}{V'''_{kid}\times K'''_{kid}})\times f_u$

Lung:

$$\frac{dX_{lu}}{dt}=Q_{lu}\times\frac{X_{cv}}{V_{cv}}-Q_{lu}\times\frac{X_{lu}}{V_{lu}}+(\frac{X'_{lu}\times PS_{lu1}}{V'_{lu}\times K'_{lu}}-\frac{PS_{lu1}\times X_{lu}}{V_{lu}}-\frac{PS_{lu3}\times X_{lu}}{V_{lu}}+\frac{X'''_{lu}\times PS_{lu3}}{V'''_{lu}\times K'''_{lu}})\times f_u$$

Intercellular stromal layer: $\frac{dX'_{lu}}{dt}=(\frac{PS_{lu1}\times X_{lu}}{V_{lu}}-\frac{X'_{lu}\times PS_{lu1}}{V'_{lu}\times K'_{lu}}-\frac{PS_{lu2}\times X'_{lu}}{V'_{lu}\times K'_{lu}}+\frac{X''_{lu}\times PS_{lu2}}{V''_{lu}\times K''_{lu}})\times f_u$

Cell layer: $\frac{dX''_{lu}}{dt}=(\frac{PS_{lu2}\times X'_{lu}}{V'_{lu}\times K'_{lu}}-\frac{X''_{lu}\times PS_{lu2}}{V''_{lu}\times K''_{lu}})\times f_u$

Erythrocyte layer: $\frac{dX'''_{lu}}{dt}=(\frac{PS_{lu3}\times X_{lu}}{V_{lu}}-\frac{X'''_{lu}\times PS_{lu3}}{V'''_{lu}\times K'''_{lu}})\times f_u$

Bone:

$$\frac{dX_{bo}}{dt}=Q_{bo}\times\frac{X_{art}}{V_{art}}-Q_{bo}\times\frac{X_{bo}}{V_{bo}}+(\frac{X'_{bo}\times PS_{bo1}}{V'_{bo}\times K'_{bo}}-\frac{PS_{bo1}\times X_{bo}}{V_{bo}}-\frac{PS_{bo3}\times X_{bo}}{V_{bo}}+\frac{X'''_{bo}\times PS_{bo3}}{V'''_{bo}\times K'''_{bo}})\times f_u$$

Intercellular stromal layer: $\frac{dX'_{bo}}{dt}=(\frac{PS_{bo1}\times X_{bo}}{V_{bo}}-\frac{X'_{bo}\times PS_{bo1}}{V'_{bo}\times K'_{bo}}-\frac{PS_{bo2}\times X'_{bo}}{V'_{bo}\times K'_{bo}}+\frac{X''_{bo}\times PS_{bo2}}{V''_{bo}\times K''_{bo}})\times f_u$

Cell layer: $\frac{dX''_{bo}}{dt}=(\frac{PS_{bo2}\times X'_{bo}}{V'_{bo}\times K'_{bo}}-\frac{X''_{bo}\times PS_{bo2}}{V''_{bo}\times K''_{bo}})\times f_u$

Erythrocyte layer: $\frac{dX'''_{bo}}{dt}=(\frac{PS_{bo3}\times X_{bo}}{V_{bo}}-\frac{X'''_{bo}\times PS_{bo3}}{V'''_{bo}\times K'''_{bo}})\times f_u$

Brain:

$$\frac{dX_{br}}{dt}=Q_{br}\times\frac{X_{art}}{V_{art}}-Q_{br}\times\frac{X_{br}}{V_{br}}+(\frac{X'_{br}\times PS_{br1}}{V'_{br}\times K'_{br}}-\frac{PS_{br1}\times X_{br}}{V_{br}}-\frac{PS_{br3}\times X_{br}}{V_{br}}+\frac{X'''_{br}\times PS_{br3}}{V'''_{br}\times K'''_{br}})\times f_u$$

Intercellular stromal layer: $\frac{dX'_{br}}{dt}=(\frac{PS_{br1}\times X_{br}}{V_{br}}-\frac{X'_{br}\times PS_{br1}}{V'_{br}\times K'_{br}}-\frac{PS_{br2}\times X'_{br}}{V'_{br}\times K'_{br}}+\frac{X''_{br}\times PS_{br2}}{V''_{br}\times K''_{br}})\times f_u$

Cell layer: $\frac{dX''_{br}}{dt}=(\frac{PS_{br2}\times X'_{br}}{V'_{br}\times K'_{br}}-\frac{X''_{br}\times PS_{br2}}{V''_{br}\times K''_{br}})\times f_u$

Erythrocyte layer: $\frac{dX'''_{br}}{dt}=(\frac{PS_{br3}\times X_{br}}{V_{br}}-\frac{X'''_{br}\times PS_{br3}}{V'''_{br}\times K'''_{br}})\times f_u$

Fat:

$$\frac{dX_{fa}}{dt}=Q_{fa}\times\frac{X_{art}}{V_{art}}-Q_{fa}\times\frac{X_{fa}}{V_{fa}}+(\frac{X'_{fa}\times PS_{fa1}}{V'_{fa}\times K'_{fa}}-\frac{PS_{fa1}\times X_{fa}}{V_{fa}}-\frac{PS_{fa3}\times X_{fa}}{V_{fa}}+\frac{X'''_{fa}\times PS_{fa3}}{V'''_{fa}\times K'''_{fa}})\times f_u$$

Intercellular stromal layer: $\frac{dX'_{fa}}{dt}=(\frac{PS_{fa1}\times X_{fa}}{V_{fa}}-\frac{X'_{fa}\times PS_{fa1}}{V'_{fa}\times K'_{fa}}-\frac{PS_{fa2}\times X'_{fa}}{V'_{fa}\times K'_{fa}}+\frac{X''_{fa}\times PS_{fa2}}{V''_{fa}\times K''_{fa}})\times f_u$

Cell layer: $\frac{dX''_{fa}}{dt}=(\frac{PS_{fa2}\times X'_{fa}}{V'_{fa}\times K'_{fa}}-\frac{X''_{fa}\times PS_{fa2}}{V''_{fa}\times K''_{fa}})\times f_u$

Erythrocyte layer: $\frac{dX'''_{fa}}{dt}=(\frac{PS_{fa3}\times X_{fa}}{V_{fa}}-\frac{X'''_{fa}\times PS_{fa3}}{V'''_{fa}\times K'''_{fa}})\times f_u$

Muscle:

$$\frac{dX_{mu}}{dt}=Q_{mu}\times\frac{X_{art}}{V_{art}}-Q_{mu}\times\frac{X_{mu}}{V_{mu}}+(\frac{X'_{mu}\times PS_{mu1}}{V'_{mu}\times K'_{mu}}-\frac{PS_{mu1}\times X_{mu}}{V_{mu}}-\frac{PS_{mu3}\times X_{mu}}{V_{mu}}+\frac{X'''_{mu}\times PS_{mu3}}{V'''_{mu}\times K'''_{mu}})\times f_u$$

Intercellular stromal layer: $\frac{dX'_{mu}}{dt}=(\frac{PS_{mu1}\times X_{mu}}{V_{mu}}-\frac{X'_{mu}\times PS_{mu1}}{V'_{mu}\times K'_{mu}}-\frac{PS_{mu2}\times X'_{mu}}{V'_{mu}\times K'_{mu}}+\frac{X''_{mu}\times PS_{mu2}}{V''_{mu}\times K''_{mu}})\times f_u$

Cell layer: $\frac{dX''_{mu}}{dt}=(\frac{PS_{mu2}\times X'_{mu}}{V'_{mu}\times K'_{mu}}-\frac{X''_{mu}\times PS_{mu2}}{V''_{mu}\times K''_{mu}})\times f_u$

Erythrocyte layer: $\frac{dX'''_{mu}}{dt}=(\frac{PS_{mu3}\times X_{mu}}{V_{mu}}-\frac{X'''_{mu}\times PS_{mu3}}{V'''_{mu}\times K'''_{mu}})\times f_u$

Skin:

$$\frac{dX_{sk}}{dt}=Q_{sk}\times\frac{X_{art}}{V_{art}}-Q_{sk}\times\frac{X_{sk}}{V_{sk}}+(\frac{X'_{sk}\times PS_{sk1}}{V'_{sk}\times K'_{sk}}-\frac{PS_{sk1}\times X_{sk}}{V_{sk}}-\frac{PS_{sk3}\times X_{sk}}{V_{sk}}+\frac{X'''_{sk}\times PS_{sk3}}{V'''_{sk}\times K'''_{sk}})\times f_u$$

Intercellular stromal layer: $\frac{dX'_{sk}}{dt}=(\frac{PS_{sk1}\times X_{sk}}{V_{sk}}-\frac{X'_{sk}\times PS_{sk1}}{V'_{sk}\times K'_{sk}}-\frac{PS_{sk2}\times X'_{sk}}{V'_{sk}\times K'_{sk}}+\frac{X''_{sk}\times PS_{sk2}}{V''_{sk}\times K''_{sk}})\times f_u$

Cell layer: $\frac{dX''_{sk}}{dt}=(\frac{PS_{sk2}\times X'_{sk}}{V'_{sk}\times K'_{sk}}-\frac{X''_{sk}\times PS_{sk2}}{V''_{sk}\times K''_{sk}})\times f_u$

Erythrocyte layer: $\frac{dX'''_{sk}}{dt}=(\frac{PS_{sk3}\times X_{sk}}{V_{sk}}-\frac{X'''_{sk}\times PS_{sk3}}{V'''_{sk}\times K'''_{sk}})\times f_u$

Gonads:

$$\frac{dX_{go}}{dt}=Q_{go}\times\frac{X_{art}}{V_{art}}-Q_{go}\times\frac{X_{go}}{V_{go}}+(\frac{X'_{go}\times PS_{go1}}{V'_{go}\times K'_{go}}-PS_{go1}\times\frac{X_{go}}{V_{go}}-\frac{PS_{go3}\times X_{go}}{V_{go}}+\frac{X'''_{go}\times PS_{go3}}{V'''_{go}\times K'''_{go}})\times f_u$$

Intercellular stromal layer: $\frac{dX'_{pa}}{dt}=(\frac{PS_{go1}\times X_{go}}{V_{go}}-\frac{X'_{go}\times PS_{go1}}{V'_{go}\times K'_{go}}-\frac{PS_{go2}\times X'_{go}}{V'_{go}\times K'_{go}}+\frac{X''_{go}\times PS_{go2}}{V''_{go}\times K''_{go}})\times f_u$

Cell layer: $\frac{dX''_{pa}}{dt}=(\frac{PS_{go2}\times X'_{go}}{V'_{go}\times K'_{go}}-\frac{X''_{go}\times PS_{go2}}{V''_{go}\times K''_{go}})\times f_u$

Erythrocyte layer: $\frac{dX'''_{pa}}{dt}=(\frac{PS_{go3}\times X_{go}}{V_{go}}-\frac{X'''_{go}\times PS_{go3}}{V'''_{go}\times K'''_{go}})\times f_u$

Heart:

$$\frac{dX_{he}}{dt}=Q_{he}\times\frac{X_{art}}{V_{art}}-Q_{he}\times\frac{X_{he}}{V_{he}}+(\frac{X'_{he}\times PS_{he1}}{V'_{he}\times K'_{he}}-\frac{PS_{he1}\times X_{he}}{V_{he}}-\frac{PS_{he3}\times X_{he}}{V_{he}}+\frac{X'''_{he}\times PS_{he3}}{V'''_{he}\times K'''_{he}})\times f_u$$

Intercellular stromal layer: $\frac{dX'_{he}}{dt}=(\frac{PS_{he1}\times X_{he}}{V_{he}}-\frac{X'_{he}\times PS_{he1}}{V'_{he}\times K'_{he}}-\frac{PS_{he2}\times X'_{he}}{V'_{he}\times K'_{he}}+\frac{X''_{he}\times PS_{he2}}{V''_{he}\times K''_{he}})\times f_u$

Cell layer: $\frac{dX''_{he}}{dt}=(\frac{PS_{he2}\times X'_{he}}{V'_{he}\times K'_{he}}-\frac{X''_{he}\times PS_{he2}}{V''_{he}\times K''_{he}})\times f_u$

Erythrocyte layer: $\frac{dX'''_{he}}{dt}=(\frac{PS_{he3}\times X_{he}}{V_{he}}-\frac{X'''_{he}\times PS_{he3}}{V'''_{he}\times K'''_{he}})\times f_u$

Arterial blood:

$$\frac{dX_{art}}{dt}=Q_{lu}\times\frac{X_{lu}}{V_{lu}}-(Q_{li}+Q_{kid}+Q_{he}+Q_{sk}+Q_{mu}+Q_{fa}+Q_{br}+Q_{bo}+Q_{go}+Q_{po})\times\frac{X_{art}}{V_{art}}+(\frac{X_{art}^{'''}\times PS_{art3}}{V_{art}^{'''}\times K_{art}^{'''}}-\frac{PS_{art3}\times X_{art}}{V_{art}})\times f_u$$

Erythrocyte layer: $\frac{dX_{art}^{'''}}{dt}=(\frac{PS_{art3}\times X_{art}}{V_{art}}-\frac{X_{art}^{'''}\times PS_{art3}}{V_{art}^{'''}\times K_{art}^{'''}})\times f_u$

Venous:

$$\frac{dX_{cv}}{dt}=Q_{he}\times\frac{X_{he}}{V_{he}}+(Q_{liv}+Q_{po})\times\frac{X_{liv}}{V_{liv}}+Q_{kid}\times\frac{X_{kid}}{V_{kid}}+Q_{sk}\times\frac{X_{sk}}{V_{sk}}+Q_{mu}\times\frac{X_{mu}}{V_{mu}}+Q_{fa}\times\frac{X_{fa}}{V_{fa}}+$$

$$Q_{br}\times\frac{X_{br}}{V_{br}}+Q_{bo}\times\frac{X_{bo}}{V_{bo}}+Q_{go}\times\frac{X_{go}}{V_{go}}-Q_{lu}\times\frac{X_{cv}}{V_{cv}}+(\frac{X_{cv}^{'''}\times PS_{cv3}}{V_{cv}^{'''}\times K_{cv}^{'''}}-\frac{PS_{cv3}\times X_{cv}}{V_{cv}})\times f_u$$

Erythrocyte layer: $\frac{dX_{cv}^{'''}}{dt}=(\frac{PS_{cv3}\times X_{cv}}{V_{cv}}-\frac{X_{cv}^{'''}\times PS_{cv3}}{V_{cv}^{'''}\times K_{cv}^{'''}})\times f_u$

Venous blood concentration=$\frac{X_{cv}}{V_{cv}}$ （mg/ml）

Note: all f_u values are identical, as they refer to the unbound fraction in plasma—i.e., the fraction of drug not bound to plasma proteins.

X represents the amount of substance, V represents the volume, Q represents the blood flow rate. PS=P*S, where P represents the permeability per unit area (cm/min), S represents the surface area, and PS is the volume permeated per unit time

$\frac{f_u}{K_{organ}^{'''}}$ is the drug unbound rate of the interstitial / cellular / erythrocyte layer of an organ,

$f_u^{'''}=\frac{f_u}{K_{organ}^{'''}}$ is the unbound rate of drugs in the red blood cell layer,

$f_u^{''}=\frac{f_u}{K_{organ}^{''}}$, is the unbound rate of drugs in the cell layer,

$f_u^{'}=\frac{f_u}{K_{organ}^{'}}$ is the unbound rate of drugs in the interstitial layer.

During drug diffusion between compartments, only unbound (free) drug molecules participate in the exchange; bound drug molecules do not. Therefore, diffusion is governed by the unbound fraction. Since PK-Sim does not explicitly include a parameter for the unbound fraction in tissues, this is instead represented via the distribution coefficient.

$$K_{organ}^{'''}=\frac{Drug\ concentration\ in\ plasma}{Drug\ concentration\ in\ the\ interstitium\ /\ cells\ /\ erythrocytes}$$

$$=\frac{\frac{Plasma\ Unbound\ Drug\ Concentration}{Plasma\ Protein\ Unbound\ Fraction}}{\frac{Interstitial/Cellular/Erythrocyte\ Unbound\ Drug\ Concentrations}{Interstitial/Cellular/Erythrocyte\ Unbound\ Fraction}}$$

$$=\frac{Plasma\ Unbound\ Drug\ Concentration}{Interstitial/Cellular/Erythrocyte\ Unbound\ Drug\ Concentrations}\times\frac{Interstitial/Cellular/Erythrocyte\ Unbound\ Fraction}{Plasma\ Protein\ Unbound\ Fraction}$$

Among them, $K^{'''}_{organ}$ denotes the distribution coefficient, representing the equilibrium concentration ratio between compartments. At equilibrium, the unbound drug concentrations in all compartments are equal, so:

$$\frac{Plasma\ Unbound\ Drug\ Concentration}{Interstitial/Cellular/Erythrocyte\ Unbound\ Drug\ Concentrations}=1$$

Thus, $K^{'''}_{organ}$ can be used to derive the unbound fraction in other compartments, enabling the estimation of free drug concentrations. (Bound drug does not participate in exchange.)

C_{Lint_K} and C_{Lint_L} represent renal or hepatic clearance, respectively, and may also be referred to as renal plasma clearance and hepatic plasma clearance.

Due to the liver clearance rate not exceeding liver blood flow, the maximum clearance rate per unit body weight can be approximately considered equal to liver blood flow. The renal clearance process is mainly determined by three parts: glomerular filtration, active secretion by renal tubules, and reabsorption by renal tubules. For compounds with active secretion, the renal clearance rate may be higher than the glomerular filtration rate, usually reaching about 2-3 times the GFR.

Species	Maximum liver clearance rate(ml/min/kg)	Maximum renal clearance rate(ml/min/kg)	References	p1 upper limit	p2 upper limit	p3 upper limit
Rats	120	30	[1][2]	0.01	0.001	0.001

Optimization algorithm: Use SciPy's differential evolution algorithm, with the following settings:

Group size=15, tolerance=0.01, mutation/crossover using best1bin strategy.

The solver uses the BDF method of solve_ivp internally, with a relative tolerance of $1e^{-3}$ and

an absolute tolerance of $1e^{-6}$.

Verification and evaluation: Use the optimal parameters to solve the smooth curve, calculate the logarithmic scale determination coefficient R^2 log of the observation time history to evaluate the goodness of fit and avoid errors dominated by high concentration points. The final parameter estimates report the optimal values and their corresponding objective function values.

In this study, the liver and kidney clearance rate parameters (C_{LintL} and C_{LintK}) and permeability (p_1, p_2, p_3) were optimized by fitting experimental blood drug concentration data using a PBPK model. The fitting process is as follows: Firstly, a multi chamber PBPK model is constructed, which includes blood and major organs/tissues. The changes in drug dosage for each organ are described by differential equations, and liver and kidney clearance rates are explicitly expressed as model parameters. Subsequently, the time series of blood drug concentrations measured experimentally was used as the fitting objective, and the logarithmic residual sum of squares was used as the optimization function. The differential evolution algorithm was employed to search for the optimal value within the given parameter range, and numerical integration was performed to solve the model. After fitting, use the optimal parameters for smooth time series simulation and calculate R^2 on a logarithmic scale to evaluate the fitting effect.

The blood flow of each organ, the volume, surface area, and distribution coefficient of each compartment in the differential equation are calculated by pksim based on the physicochemical properties of the compound and the weight and species of the animal. The physicochemical properties of the compound were obtained from Drugbank.

References:

[1]Gibson CR, Gleason A, Messina E. Measurement of total liver blood flow in intact anesthetized rats using ultrasound imaging. Pharmacol Res Perspect. 2021;9(2):e00731. doi:10.1002/prp2.731

[2]Davies B, Morris T. Physiological parameters in laboratory animals and humans. Pharm Res. 1993;10(7):1093-5. doi: 10.1023/A:1018943613122.
